# Vocational self-stigma and problematic smartphone use: self-regulatory fatigue as an indirect pathway and professional identity as a second-stage moderator

**DOI:** 10.3389/fpsyg.2026.1942617

**Published:** 2026-10-15

**Authors:** Yong Chen, Xiaoxian Zheng, Yingping Yan

**Affiliations:** Zhejiang Tourism and Health College, Zhoushan, China

**Keywords:** moderated mediation, problematic smartphone use, professional identity, self-regulatory fatigue, self-stigma, vocational education

## Abstract

Problematic smartphone use is common among young people, and research on its antecedents has focused mainly on individual dispositions and transient emotional states. Less is known about whether self-stigma, a chronic and group-based devaluation of the self, is linked to problematic smartphone use through self-regulatory fatigue, and whether professional identity weakens that link. We examined these questions in a cross-sectional survey of 1,352 students at Chinese higher vocational colleges, whose educational track carries a persistent status penalty. Vocational self-stigma was associated with greater self-regulatory fatigue, which in turn was associated with more problematic smartphone use [indirect effect = 0.127, 95% CI (0.099, 0.157)]. This indirect association was larger when measurement error was modeled, remained significant across alternative scale compositions, covariate sets, and analytic samples, and was smaller but still significant when differences between cities of response were held constant. Professional identity moderated the second stage of the pathway: the association between fatigue and problematic use weakened as professional identity increased [*B* = −0.142, 95% CI (−0.228, −0.053)] but remained significant for nearly all students, including those with a strong professional identity. The corresponding first-stage interaction was close to zero [*B* = −0.008, 95% CI (−0.068, 0.053)]. Items reflecting awareness of public devaluation and items reflecting self-devaluation showed similar indirect associations. The findings are consistent with a view of vocational self-stigma as a source of regulatory strain and suggest that professional identity is related to how students act under that strain more than to how closely self-stigma is tied to it. Longitudinal and intervention studies are needed to establish the temporal order of these associations.

## 1 Introduction

Problematic smartphone use refers to smartphone engagement that is difficult to control and that interferes with study, sleep, work, or relationships (Billieux et al., 2015; Elhai et al., 2017). The term is preferred here to “smartphone addiction” because the status of the behavior as an addictive disorder remains contested (Panova and Carbonell, 2018), although its costs are well documented. Its prevalence among young people has risen over the past decade (Lu et al., 2024), and it co-occurs with depression, anxiety, stress, and poor sleep (Sohn et al., 2019).

Most research on why some young people develop this pattern has examined qualities of the person and states of the moment. Weak self-regulation and low self-control are among the most consistent predictors (Yang et al., 2019; Zhao et al., 2024), and academic anxiety and intolerance of uncertainty are linked to problematic use through self-regulatory fatigue (Wang et al., 2024; Kong et al., 2025). Higher self-esteem is associated with fewer symptoms of mobile phone addiction (Leung, 2008), social phobia is linked to problematic use through lower self-esteem, and low life satisfaction is linked to it through psychological distress and fear of missing out (Soraci et al., 2025, 2026). Common to these accounts is the idea that smartphone use helps people manage discomfort, which the compensatory internet use model makes explicit (Kardefelt-Winther, 2014) and which the Interaction of Person-Affect-Cognition-Execution (I-PACE) model links to weakened inhibitory control (Brand et al., 2019). Discomfort that originates in a person’s social position has received far less attention, although a devalued group membership cannot simply be set aside and follows people across situations.

Stigma, one prominent source of such discomfort, arises when labeling, stereotyping, separation, status loss, and discrimination converge under conditions of unequal power (Link and Phelan, 2001). Its psychological consequences depend on how people appraise and cope with the threats it poses to their identity (Major and O’Brien, 2005). Coping with these threats taxes self-regulation, and being the target of prejudice impairs self-control in domains unrelated to the prejudice itself (Inzlicht et al., 2006; Genç Turhan and Kuşdil, 2025). Perceived discrimination has also been linked to problematic smartphone use, for example among deaf and hard-of-hearing students through a diminished sense of security and social avoidance (Guan et al., 2023). Stigma may therefore be linked to problematic smartphone use through the same regulatory route documented for anxiety and uncertainty, with the difference that it is chronic and anchored in social structure.

Students in Chinese higher vocational colleges are a population for whom this possibility is especially relevant. In China, vocational education is widely regarded as a compensatory track that ranks below academic education (Guan and Blair, 2024). Admission to higher education is governed by the national college entrance examination, and students whose scores fall short of undergraduate programs are commonly channeled into vocational colleges (Ye et al., 2026). Vocational students are accordingly cast as academic failures, a stereotype reproduced in everyday interaction and within vocational colleges themselves (Ling, 2015; Wang, 2021; Bailey et al., 2024) and measurable even among the junior high school students who may later enter vocational schools (Wang et al., 2022). The penalty persists even though the revised Vocational Education Law declares vocational and general education to be of equal importance (National People’s Congress, 2022), and the lower standing of vocational tracks and of the occupations they serve has been documented across many national systems (Fuller and Unwin, 2011; Billett et al., 2022). Students in technical and vocational tracks report being looked down upon because of their program, a perception that predicts a sense of futility beyond ability and social background (Spruyt et al., 2015). Among Chinese higher vocational students, perceived stigma is associated with greater self-stigma and weaker identification with vocational education (Cui and Zhao, 2025), final-year students describe a strong wish to escape the vocational identity (Liu et al., 2026), and graduates describe exclusion from opportunities and doubts about their own competence (Liu, 2025).

Research with this population has begun to connect the low standing of vocational education to smartphone use. Among Chinese higher vocational students, perceived discrimination is associated with mobile phone addiction through negative emotions and learning burnout (Li W. et al., 2024). Two questions remain open. One is whether devaluation matters only as a perception of how others treat vocational students or also as a judgment that students apply to themselves. The other is whether the association also runs through self-regulatory fatigue, which chronic identity threat would be expected to produce, in addition to affective and motivational states. A regulatory account raises a further, practical question: whether personal resources that students bring to their studies weaken the pathway, and at which stage.

The present study addresses these questions in a large sample of Chinese higher vocational students. We examined whether self-regulatory fatigue accounts for the association between vocational self-stigma and problematic smartphone use, and whether professional identity, a clear and valued sense of one’s future occupation, moderates the path from self-stigma to fatigue, the path from fatigue to problematic use, or both.

## 2 Theoretical framework and hypotheses

Self-stigma refers to the process through which members of a devalued group turn public stereotypes against themselves. In the staged model developed by Corrigan and colleagues, people first become aware of the stereotypes held about their group, then come to agree with them, and finally apply them to themselves, with lower self-esteem and self-efficacy as the consequence (Corrigan et al., 2009; Corrigan and Rao, 2012). Stereotype awareness is the entry point of this process, although it is often distinguished from self-stigma proper and treated as perceived stigma (Corrigan et al., 2006). It overlaps with stigma consciousness, the expectation of being judged through the lens of a stereotype about one’s group (Pinel, 1999), and self-application corresponds to the internalized devaluation of the self. Field research in a Chinese secondary vocational school has described how this process unfolds: students first meet the devaluation of vocational education in their families and, after enrollment, gradually apply it to themselves while both resisting and accommodating it (Chen and Du, 2022). A study of Chinese higher vocational students measured perceived stigma and self-stigma separately and found the former to be associated with greater self-stigma (Cui and Zhao, 2025). Instruments developed for Chinese vocational students combine the two, pairing perceptions of society’s negative evaluation with self-devaluation and its consequences (Leng et al., 2025; Yan, 2025). Following these instruments, we use vocational self-stigma for a composite that spans awareness of public devaluation and its application to the self, and we examine the two components separately where the distinction matters. Public stigma, the attitudes held by others, was not measured.

Vocational self-stigma is likely to tax self-regulation. The identity threat model holds that people with a stigmatized identity repeatedly appraise whether situations threaten that identity and whether they can cope, and that threat appraisals set off involuntary stress responses along with effortful coping (Major and O’Brien, 2005). The cues that prompt these appraisals recur wherever the identity is known or relevant, and so do the demands on self-regulation. Stereotype threat taxes executive functions such as working memory and the suppression of unwanted thoughts and feelings (Schmader et al., 2008), and academic stereotype threat has been documented among Chinese higher vocational students (Bao et al., 2023). Members of subordinate groups show impaired self-control after being targeted by prejudice, after witnessing bias, and when anticipating it (Inzlicht et al., 2006; Genç Turhan and Kuşdil, 2025). These costs do not require overt mistreatment: structural stigma places people in positions of low power, which heighten vigilance and behavioral inhibition (Richman and Lattanner, 2014), and difficulties in regulating emotion have been proposed as a general pathway through which stigma harms health (Hatzenbuehler, 2009).

The strength model, which treats self-control as drawing on a limited resource, supplied much of the original rationale for linking stigma to impaired self-control (Baumeister and Vohs, 2016). Its central laboratory effect has proved difficult to reproduce. A bias-corrected meta-analysis and a large multilaboratory replication found little evidence of acute depletion in the sequential-task paradigm (Carter et al., 2015; Hagger et al., 2016), prompting calls for a new research agenda (Dang and Hagger, 2019), and later multilaboratory projects found a small effect in one case and evidence favoring the null in another (Dang et al., 2021; Vohs et al., 2021). This body of work concerns a brief manipulation followed immediately by a second task. Self-regulatory fatigue, as assessed by questionnaire, refers instead to an enduring state of diminished cognitive, emotional, and behavioral self-regulatory capacity. The construct was developed with people living with chronic illness, whose regulatory demands persist for months or years, and it is related to, yet distinct from, trait self-control and general fatigue (Solberg Nes et al., 2013). The chronic demands that define it resemble those imposed by a lasting devalued identity, so vocational self-stigma should be associated with greater self-regulatory fatigue.

Self-regulatory fatigue has direct implications for smartphone use. The smartphone offers immediate reward and relief at almost any moment, and in everyday life, desires for media use are among those most likely to prevail over attempts at self-control (Hofmann et al., 2012). The compensatory internet use model locates the problem in the use of online activity to escape difficulties and relieve negative mood (Kardefelt-Winther, 2014), and the I-PACE model specifies how cue-elicited craving, combined with weakened inhibitory control, fosters habitual use (Brand et al., 2019). Under regulatory strain, the motive to seek relief grows while the capacity to stop declines. In line with this reasoning, self-regulatory fatigue accounts for part of the associations of academic anxiety and intolerance of uncertainty with problematic smartphone use (Wang et al., 2024; Kong et al., 2025).

*H1*: Vocational self-stigma is positively associated with problematic smartphone use.

*H2*: Self-regulatory fatigue mediates this association, such that vocational self-stigma is positively associated with self-regulatory fatigue, which in turn is positively associated with problematic smartphone use.

Whether a valued identity can weaken this pathway depends partly on what the identity is attached to. Research on the social cure shows that group memberships can protect health by providing belonging, meaning, and support (Haslam et al., 2018), and identification with a devalued group can help people withstand discrimination, particularly when it is one of several valued identities (Koc and Hausmann, 2025). Identification with the stigmatized group itself can also backfire: among prisoners, ties to fellow inmates were associated with less distress, but the centrality of the prisoner identity was associated with more self-stigma and perceived exclusion (Traversa et al., 2026), and in weight stigma research the direction of effects depends on which facet of identification is measured (Hudson et al., 2025).

These findings concern identification with the stigmatized category itself, whereas professional identity is directed at a valued occupational future. Vocational identity has been conceptualized as a stable psychological attribute, as a socially constructed and evolving sense of self, and as a career-related construct, and the literature uses vocational, professional, occupational, and career identity largely interchangeably (Kryger and Qvortrup, 2025). We adopt the career-related view, in which vocational identity denotes a clear and stable picture of one’s goals, interests, and talents (Holland et al., 1980), because it matches the content of the instrument used here and concerns occupational goals that can compete with smartphone use. We use the term professional identity for students’ endorsement of their field of study, their sense of fit with it, their commitment to a related occupation, and the clarity of their occupational direction. Professional identity develops alongside students’ self-esteem during vocational study (Li M. et al., 2024). It is nonetheless exposed to devaluation: among Chinese vocational students, self-stigma and stereotype threat are both associated with weaker professional or career identity (Leng et al., 2025; Ye et al., 2026), and social narratives about vocational education shape how such identities form (Kryger and Qvortrup, 2025).

A strong professional identity may weaken both stages of the pathway. The identity threat model holds that the resources people bring to a threatening situation shape whether they appraise it as exceeding their capacity to cope (Major and O’Brien, 2005), and affirming a valued domain of the self can blunt defensive and stress responses to threat (Cohen and Sherman, 2014). A clear and valued occupational future could serve as such a resource, so that self-stigma would weigh less heavily on the self-regulation of students who have one. At the second stage, students with a clear occupational direction hold goals that compete with the smartphone for time and attention, and online escape offers them less (Kardefelt-Winther, 2014; Brand et al., 2019). The two stages carry different implications. Moderation of the first would suggest that professional identity shapes how closely self-stigma is tied to regulatory strain, and moderation of the second that it shapes how the strain is expressed in behavior. The distinction indicates whether building professional identity might reduce the regulatory cost of vocational self-stigma or mainly alter its behavioral expression.

*H3*: Professional identity moderates the association between vocational self-stigma and self-regulatory fatigue, such that the association is weaker at higher levels of professional identity.

*H4*: Professional identity moderates the association between self-regulatory fatigue and problematic smartphone use, such that the association is weaker at higher levels of professional identity.

## 3 Materials and methods

### 3.1 Participants and procedure

Data were collected on 2 and 3 April 2026 through an online questionnaire administered at five higher vocational colleges, using convenience sampling combined with cluster sampling. Most respondents (1,236, 91.4%) opened the questionnaire link through WeChat. Participation was voluntary and anonymous, and no compensation was offered. The study was approved by the Ethics Committee of Zhejiang Tourism and Health College, and all participants gave informed consent electronically after being informed of the study’s purpose, the anonymity of their responses, and their right to withdraw at any point without consequence.

Of the 1,398 questionnaires returned, 46 were removed for implausibly short completion times, invariant response patterns, or missing responses on the study variables, leaving an analytic sample of 1,352 (96.7%) with no missing data. The thresholds applied in this initial screening were not recorded, and only the 1,352 retained questionnaires were available for the present analyses, so data quality was examined further with explicit criteria in sensitivity analyses.

The analytic sample comprised 501 male (37.1%) and 851 female (62.9%) students; 1,065 (78.8%) were in their first year, 268 (19.8%) in their second, and 19 (1.4%) in their third. Of these students, 395 (29.2%) were only children, and 254 (18.8%), 260 (19.2%), and 838 (62.0%) came from urban areas, county towns, and rural areas, respectively. Family economic circumstances were self-rated as poor by 159 (11.8%), average by 1,117 (82.6%), and good by 76 (5.6%). Network addresses placed 1,066 responses (78.8%) in Zhejiang province, mainly in Hangzhou (*n* = 380), Ningbo (*n* = 366), and Zhoushan (*n* = 291), and 286 elsewhere, principally in Ningxia (*n* = 158), Hubei (*n* = 86), and Jiangxi (*n* = 38). Network location indicates where the questionnaire was completed, not necessarily a student’s college or home region, and was used to distinguish response contexts.

The questionnaire comprised 30 items: 8 background questions and the 22 scale items. The median completion time was 99 s (*M* = 134 s, range 28 to 5,460 s), and 212 respondents (15.7%) finished in under 60 s, that is, in less than about 2 s per item, a common indicator of insufficient effort (Huang et al., 2012). Identical answers to all 22 scale items were rare (10 respondents, 0.7%), whereas identical answers within a single scale were common, ranging from 22.8% of respondents on the self-stigma and fatigue items to 29.8% on the problematic smartphone use items. On the professional identity scale, 381 respondents (28.2%) chose the midpoint for every item. These respondents did not finish faster than others (median 100 s vs. 99 s), but inattentive responses tend to cluster at the scale midpoint (Huang et al., 2015), and a moderator concentrated at a single value can distort interaction estimates. Respondents who answered every scale item identically, those who finished in under 60 s, and the midpoint responders were therefore each removed in a separate sensitivity analysis (Section 4.4).

### 3.2 Measures

All items were rated on a five-point scale from 1 (*strongly disagree*) to 5 (*strongly agree*), and scale scores were computed as item means. Because the questionnaire was administered alongside other institutional assessments, each construct was measured with a short form whose items were selected from the full instrument for their content before data collection; for problematic smartphone use and professional identity, every dimension of the source instrument is represented. Supplementary Table S1 lists each item and its content domain. The self-stigma and professional identity instruments were validated in Chinese dissertations, so their structure in the present sample was examined directly (Section 4.1).

#### 3.2.1 Vocational self-stigma

Five items were taken from the Self-Stigma Scale for Vocational School Students (Yan, 2025), which was developed with Chinese vocational students. Two items assess awareness of public devaluation (e.g., believing that many people in society are prejudiced against higher vocational students), two assess self-devaluation (e.g., feeling less capable than students at general universities), and one assesses concealment (reluctance to mention one’s college to others). The assignment of these items to the dimensions of the source instrument was not recorded, so they are described here by their content. The concealment item, which describes a coping response, was also analyzed on its own. Higher scores indicate stronger vocational self-stigma. Cronbach’s *α* = 0.886.

#### 3.2.2 Self-regulatory fatigue

Five items were taken from the 16-item Chinese version of the Self-Regulatory Fatigue Scale (Wang et al., 2015), an adaptation of the 18-item instrument of Solberg Nes et al. (2013) that preserves its cognitive, emotional, and behavioral components. The items describe difficulty sustaining attention, lacking the energy to see tasks through, abandoning tasks partway, failing to persist despite intending to, and, reverse-scored, being self-disciplined in daily life. Cronbach’s *α* = 0.872.

#### 3.2.3 Problematic smartphone use

Six items were taken from the 17-item Mobile Phone Addiction Index (Leung, 2008) and represent its four symptom dimensions: loss of control (use exceeding intended limits and unsuccessful attempts to cut down), anxiety and craving (discomfort when not using the phone and compulsive checking), withdrawal and escape (using the phone to avoid problems), and productivity loss (interference with study and daily life). Cronbach’s *α* = 0.900.

#### 3.2.4 Professional identity

Six items were taken from the Vocational Identity Scale for college students (Yuan, 2008), which covers vocational interest, appraisal of one’s own ability, career decision, and the stability of occupational goals. The items concern liking and valuing one’s field, belief in one’s fit with a related occupation, commitment to such an occupation, and clarity of occupational direction; two items describing confusion about future employment and uncertainty about one’s fit with the field were reverse-scored. Higher scores indicate a clearer, more stable, and more valued professional identity. Cronbach’s *α* = 0.898. This instrument is distinct from the Vocational Identity Status Assessment validated for Chinese technical college students by Zhang et al. (2019), which indexes identity process and status rather than identity content.

#### 3.2.5 Covariates

Sex and year of study were included in all models. Both are demographic characteristics that cannot be consequences of the study variables, and sex differences in the prevalence of problematic smartphone use have been documented (Lu et al., 2024). In preliminary comparisons, sex was associated with self-stigma, problematic use, and professional identity, and year of study with self-stigma and fatigue, whereas only-child status, home location, and family economic circumstances were unrelated to fatigue and problematic use (all *p* > 0.25). Daily smartphone use time, clarity of career planning, and self-rated academic performance describe behavior and development that overlap with the study constructs: use time and career planning parallel problematic use and professional identity, and academic performance may itself reflect fatigue and problematic use. Adjusting for them in the primary models could therefore remove part of the associations of interest, so these three variables and family economic circumstances were added in a sensitivity analysis. Response location was examined in separate sensitivity analyses because it is only a proxy for institutional and regional context.

### 3.3 Data analysis

Confirmatory factor analysis compared the hypothesized four-factor model with more parsimonious alternatives and with a five-factor model that separated the two awareness items from the other three self-stigma items. Models were estimated by maximum likelihood (the default Wishart likelihood in semopy) with item responses treated as continuous. Because multivariate normality could not be assumed (Mardia, 1970), all models were also estimated with robust maximum likelihood (MLR), and the hypothesized and five-factor models were additionally estimated with the items treated as ordinal (WLSMV), a combination that allows five-category items to be analyzed with confidence (Rhemtulla et al., 2012). The four- and five-factor models were compared with the scaled χ^2^ difference tests appropriate to each estimator (Satorra, 2000; Satorra and Bentler, 2001). Model fit was evaluated with the comparative fit index (CFI), Tucker-Lewis index (TLI), root mean square error of approximation (RMSEA), and standardized root mean square residual (SRMR), and competing models were compared with the Akaike and Bayesian information criteria (AIC and BIC). Convergent validity was assessed with standardized loadings, average variance extracted (AVE), composite reliability, Cronbach’s alpha, and McDonald’s omega, and discriminant validity by comparing the square root of each construct’s AVE with its correlations with the other constructs and with the heterotrait-monotrait ratio (HTMT). Because all measures were self-reported on a single occasion, we compared the four-factor model with a single-factor model and inspected the first unrotated component of all items, recognizing that neither procedure can rule out common method variance (Podsakoff et al., 2024).

Mediation and moderated mediation were estimated with ordinary least squares regression following Hayes (2022), corresponding to PROCESS Models 4 and 59. In Model 59, professional identity moderates the path from self-stigma to fatigue (the first stage), the path from fatigue to problematic use (the second stage), and the direct path (Edwards and Lambert, 2007). Predictor, mediator, and moderator were mean-centered before forming product terms. Breusch-Pagan tests indicated heteroskedastic residuals in both equations (both *p* < 0.001), so regression coefficients were tested with HC3 heteroskedasticity-consistent standard errors (Hayes and Cai, 2007), which were also used in the Johnson-Neyman analysis of the conditional effect of fatigue across the range of professional identity. Indirect effects, conditional indirect effects, and interaction coefficients were tested with percentile bootstrap confidence intervals based on 5,000 resamples, and all interactions were tested two-sided. Because professional identity enters both stages of Model 59, the indirect effect is a quadratic function of the moderator, so the index of linear moderated mediation does not apply (Hayes, 2015); conditional indirect effects at 1 SD below and above the mean of professional identity were compared directly instead. The indirect effect and its confidence interval served as the primary index of mediation. The proportion mediated is reported only descriptively, because it is unstable when direct and indirect effects are small or opposite in sign (Preacher and Kelley, 2011) and, with cross-sectional data, does not indicate what share of a causal effect is transmitted through fatigue. Standardized coefficients were obtained by rescaling unstandardized estimates by the sample standard deviations.

To judge whether a nonsignificant first-stage interaction reflected a negligible effect or imprecision, its 95% confidence interval was compared with equivalence bounds (Lakens, 2017), a stricter criterion than the 90% interval that corresponds to two one-sided tests at *α* = 0.05. The bounds were anchored on the moderation observed at the second stage. They mark the first-stage interaction that would change the path from self-stigma to fatigue across ±1 SD of professional identity by the same proportion as the observed second-stage interaction changed the path from fatigue to problematic use. As a benchmark internal to the data, they were applied only to the full-sample model.

A latent-variable version of the mediation model, with sex and year of study as covariates of fatigue and problematic use, estimated the paths free of measurement error. The observed-variable models were retained as the primary analysis because the scales were highly reliable, which limits attenuation, and because conditional indirect effects and Johnson-Neyman intervals are well established in the regression framework, whereas latent interaction models require additional distributional assumptions. Measurement error attenuates product terms more than main effects, but given the reliabilities and intercorrelations in Table 1, the reliability of both interaction terms is about 0.80 (Aiken and West, 1991), so attenuation would affect the first- and second-stage interactions to a similar degree.

**Table 1:** Descriptive statistics, reliability, and correlations (*N* = 1,352).

Variable	*M*	*SD*	*α*	*ω*	AVE	1	2	3	4
1. Vocational self-stigma	2.833	0.932	0.886	0.886	0.610	(0.781)			
2. Self-regulatory fatigue	2.793	0.726	0.872	0.873	0.579	0.351	(0.761)		
3. Problematic smartphone use	2.815	0.768	0.900	0.901	0.603	0.297	0.492	(0.776)	
4. Professional identity	3.083	0.691	0.898	0.900	0.601	−0.134	−0.242	−0.253	(0.775)

All correlations *p* < 0.001. Values in parentheses on the diagonal are the square root of the average variance extracted. *α*, Cronbach’s alpha; *ω*, McDonald’s omega; AVE, average variance extracted.

Robustness was examined in eleven alternative specifications, which, together with the primary model, form the twelve analyses summarized in Table 2 and described in Supplementary Table S2. Each specification varied one feature of the primary model: the composition of the scales (omitting the energy-related fatigue item F3, the only item representing the emotional facet, or restricting the self-stigma scale to its self-devaluation or awareness items), the covariate set (adding the four further covariates, or adding response location), or the analytic sample (removing invariant responders, respondents who finished in under 60 s, or midpoint responders on professional identity, or restricting the sample to first-year students or to either response location). The concealment item was also examined on its own, and a model with the order of self-stigma and problematic smartphone use reversed was estimated for comparison. Because institutional membership was not recorded, the primary model was also re-estimated with fixed effects for the city from which each questionnaire was submitted, which absorb mean differences between response contexts. Descriptive statistics and the principal component analysis were computed in SPSS 27.0; the regression, bootstrap, and Johnson-Neyman analyses were run in Python 3.12 (statsmodels 0.15.0), the maximum likelihood confirmatory factor analyses in semopy 2.3.11, and the robust and ordinal confirmatory factor analyses in R 4.3.3 (lavaan 0.6-17 and psych 2.4.1; Rosseel, 2012).

**Table 2:** Indirect effect and interaction coefficients across twelve analytic specifications.

Specification	*n*	*a*	*b*	Indirect effect [95% CI]	*a*_3_ [95% CI]	*b*_3_ [95% CI]
Primary model	1,352	0.271	0.468	0.127 [0.099, 0.157]	−0.008 [−0.068, 0.053]	−0.142 [−0.228, −0.053]
Fatigue scale without energy item	1,352	0.278	0.456	0.127 [0.098, 0.156]	−0.004 [−0.068, 0.062]	−0.131 [−0.216, −0.046]
Self-stigma, self-devaluation items only	1,352	0.229	0.479	0.110 [0.086, 0.135]	−0.001 [−0.052, 0.053]	−0.127 [−0.216, −0.037]
Self-stigma, awareness items only	1,352	0.232	0.479	0.111 [0.084, 0.139]	−0.012 [−0.071, 0.048]	−0.160 [−0.240, −0.075]
Extended covariate set	1,352	0.245	0.444	0.109 [0.082, 0.138]	0.001 [−0.062, 0.066]	−0.144 [−0.232, −0.055]
Response location added as covariate	1,352	0.210	0.411	0.086 [0.063, 0.111]	0.007 [−0.046, 0.060]	−0.120 [−0.205, −0.032]
Invariant responders removed	1,342	0.271	0.468	0.127 [0.099, 0.155]	−0.008 [−0.070, 0.053]	−0.142 [−0.227, −0.049]
Completion time ≥ 60 s	1,140	0.271	0.471	0.128 [0.098, 0.160]	0.023 [−0.047, 0.092]	−0.141 [−0.231, −0.046]
First-year students only	1,065	0.283	0.459	0.130 [0.100, 0.162]	0.019 [−0.052, 0.088]	−0.133 [−0.231, −0.035]
Zhejiang respondents	1,066	0.164	0.395	0.065 [0.041, 0.092]	0.014 [−0.068, 0.104]	−0.108 [−0.215, 0.015]
Respondents outside Zhejiang	286	0.256	0.373	0.095 [0.050, 0.147]	0.039 [−0.067, 0.152]	−0.249 [−0.402, −0.086]
Midpoint responders removed	971	0.311	0.515	0.160 [0.124, 0.196]	−0.014 [−0.074, 0.047]	−0.152 [−0.241, −0.066]

a, path from self-stigma to fatigue; b, path from fatigue to problematic smartphone use; a_3_, first-stage interaction (self-stigma × professional identity); b_3_, second-stage interaction (fatigue × professional identity). Confidence intervals are percentile bootstrap intervals based on 5,000 resamples. With HC3 standard errors, *p* values for a_3_ ranged from 0.486 to 0.985, and those for b_3_ were below 0.01 in every specification except the Zhejiang subsample (*p* = 0.072). Supplementary Table S2 describes each specification.

## 4 Results

### 4.1 Preliminary analyses

The hypothesized four-factor model fit the data closely, *χ*^2^(203) = 353.08, *χ*^2^/*df* = 1.74, CFI = 0.991, TLI = 0.989, RMSEA = 0.023, SRMR = 0.019. Every more parsimonious alternative fit substantially worse (Table 3). Merging self-regulatory fatigue with problematic smartphone use, the pair most likely to be confounded, reduced CFI to 0.872 and raised RMSEA to 0.086; the single-factor model reached CFI = 0.458. Both information criteria favored the hypothesized model over every alternative. Item skewness ranged from −0.30 to 0.24 and kurtosis from −0.67 to 1.36, but multivariate kurtosis was elevated (Mardia’s *z* = 102.24, *p* < 0.001). Robust estimation supported the same conclusions: with MLR, the four-factor model yielded a scaled *χ*^2^(203) = 306.75, CFI = 0.993, TLI = 0.991, RMSEA = 0.021, and every alternative again fit substantially worse; with WLSMV, it yielded *χ*^2^(203) = 392.86, CFI = 0.995, TLI = 0.995, RMSEA = 0.026, with standardized loadings from 0.743 to 0.856.

**Table 3:** Fit of alternative measurement models (*N* = 1,352).

Model	*χ* ^2^	*df*	χ^2^/*df*	CFI	TLI	RMSEA	SRMR	ΔAIC	ΔBIC
Four-factor (hypothesized)	353.08	203	1.74	0.991	0.989	0.023	0.019	0.00	0.00
Five-factor (self-stigma separated)	351.71	199	1.77	0.990	0.989	0.024	0.019	6.63	27.47
Three-factor (fatigue and use combined)	2266.32	206	11.00	0.872	0.856	0.086	0.070	1907.24	1891.61
Three-factor (self-stigma and identity combined)	4639.16	206	22.52	0.724	0.691	0.126	0.149	4280.08	4264.45
Two-factor (self-stigma with identity; fatigue with use)	6549.85	208	31.49	0.605	0.562	0.150	0.164	6186.77	6160.72
One-factor	8914.14	209	42.65	0.458	0.401	0.176	0.166	8549.06	8517.80

Models were estimated by maximum likelihood; results with robust maximum likelihood (MLR) and with the items treated as ordinal (WLSMV) are reported in Section 4.1. The five-factor model separates the two awareness items from the other three self-stigma items; its comparison with the four-factor model gives Δ*χ*^2^(4) = 1.37, *p* = 0.849. The two-factor model combines self-stigma with professional identity and self-regulatory fatigue with problematic smartphone use. ΔAIC and ΔBIC are differences from the hypothesized model; positive values favor the hypothesized model. CFI, comparative fit index; TLI, Tucker-Lewis index; RMSEA, root mean square error of approximation; SRMR, standardized root mean square residual; AIC, Akaike information criterion; BIC, Bayesian information criterion.

Separating the two awareness items from the other three self-stigma items did not improve fit, Δ*χ*^2^(4) = 1.37, *p* = 0.849, a result that held with the scaled difference tests (MLR, Δ*χ*^2^(4) = 1.16, *p* = 0.885; WLSMV, Δ*χ*^2^(4) = 2.49, *p* = 0.646). The estimated latent correlation between the two factors was 1.00 (1.01 with WLSMV), and the corresponding composite scores correlated at *r* = 0.779, so self-stigma was treated as a single factor.

Standardized loadings ranged from 0.709 to 0.822 for vocational self-stigma, 0.736 to 0.791 for self-regulatory fatigue, 0.745 to 0.793 for problematic smartphone use, and 0.744 to 0.812 for professional identity, all *p* < 0.001. Average variance extracted was 0.610, 0.579, 0.603, and 0.601 respectively, and composite reliability was 0.886, 0.873, 0.901, and 0.900. The square root of average variance extracted exceeded every corresponding inter-construct correlation for all four constructs. Heterotrait-monotrait ratios were 0.399 for self-stigma with fatigue, 0.332 for self-stigma with problematic use, 0.553 for fatigue with problematic use, and below 0.280 in absolute value for all pairings involving professional identity. The first unrotated component of a principal component analysis of all 22 items accounted for 31.96% of the variance, and four components exceeded an eigenvalue of one, together explaining 67.23%. Together with the poor fit of the single-factor model, these results indicate that the four constructs were empirically distinguishable, although they cannot exclude common method variance, which may inflate or deflate observed associations (Podsakoff et al., 2024).

Means, standard deviations, reliabilities, and correlations appear in Table 1. All associations were in the expected direction and significant at *p* < 0.001. Vocational self-stigma correlated with self-regulatory fatigue at *r* = 0.351 and with problematic smartphone use at *r* = 0.297. Self-regulatory fatigue correlated with problematic smartphone use at *r* = 0.492. Professional identity correlated negatively with all three, most strongly with problematic smartphone use, *r* = −0.253.

### 4.2 Indirect association through self-regulatory fatigue

Controlling for sex and year of study, vocational self-stigma was positively associated with problematic smartphone use, *B* = 0.240, *SE* = 0.026, *t* = 9.33, *p* < 0.001, *R*^2^ = 0.092, supporting H1. Self-stigma was also positively associated with self-regulatory fatigue, *B* = 0.271, *SE* = 0.024, *t* = 11.22, *p* < 0.001, *R*^2^ = 0.128. When fatigue was added to the model, it was positively associated with problematic use, *B* = 0.468, *SE* = 0.036, *t* = 13.18, *p* < 0.001, and the direct association of self-stigma with problematic use remained significant, *B* = 0.113, *SE* = 0.024, *t* = 4.69, *p* < 0.001, *R*^2^ = 0.263.

The indirect effect was 0.127, bootstrap *SE* = 0.015, 95% CI [0.099, 0.157], or β = 0.154 against a standardized total association of 0.291, supporting H2. Descriptively, the indirect effect amounted to 52.8% of the total association. The latent-variable model fit well, χ^2^(132) = 241.99, CFI = 0.990, TLI = 0.989, RMSEA = 0.025, SRMR = 0.026, with standardized paths of 0.395 from self-stigma to fatigue, 0.505 from fatigue to problematic use, and 0.124 for the direct path, and a standardized indirect effect of 0.199.

### 4.3 Moderation by professional identity

Table 4 reports the moderated mediation model, and Figure 1 displays its central paths. In the equation for self-regulatory fatigue, vocational self-stigma was positively associated with fatigue, *B* = 0.251, *t* = 10.50, *p* < 0.001, and professional identity was negatively associated with it, *B* = −0.207, *t* = −6.42, *p* < 0.001. The interaction between self-stigma and professional identity was close to zero, *B* = −0.008, *SE* = 0.032, *t* = −0.26, *p* = 0.793, bootstrap 95% CI [−0.068, 0.053]. H3 was not supported. The 95% confidence interval lay entirely within the equivalence bounds of ±0.080, so a first-stage interaction as large, in proportional terms, as the observed second-stage interaction can be rejected for the full sample.

**Table 4:** Moderated mediation model predicting self-regulatory fatigue and problematic smartphone use (*N* = 1,352).

Predictor	Self-regulatory fatigue				Problematic smartphone use			
	*B*	*SE*	*β*	*t*	*B*	*SE*	*β*	*t*
Vocational self-stigma	0.251	0.024	0.32	10.50***	0.104	0.023	0.13	4.45***
Self-regulatory fatigue					0.444	0.035	0.42	12.79***
Professional identity	−0.207	0.032	−0.20	−6.42***	−0.139	0.032	−0.13	−4.35***
Self-stigma × identity	−0.008	0.032	−0.01	−0.26	−0.026	0.035	−0.02	−0.74
Fatigue × identity					−0.142	0.046	−0.09	−3.11**
Sex	−0.021	0.041	−0.01	−0.50	0.100	0.040	0.06	2.49*
Year of study	−0.094	0.041	−0.06	−2.30*	0.011	0.039	0.01	0.29
*R* ^2^	0.166				0.295			
*F*	41.12***				62.59***			

Identity, professional identity. Predictor, mediator, and moderator were mean-centered before forming product terms. Standard errors, *t* values, and *F* tests are based on HC3 heteroskedasticity-consistent estimates. *F* degrees of freedom were (5, 1,346) and (7, 1,344). *β* denotes coefficients rescaled by the sample standard deviations of the variables involved. Sex coded 1 = male, 2 = female. *p < 0.05, **p < 0.01, ***p < 0.001.

**Figure 1:**
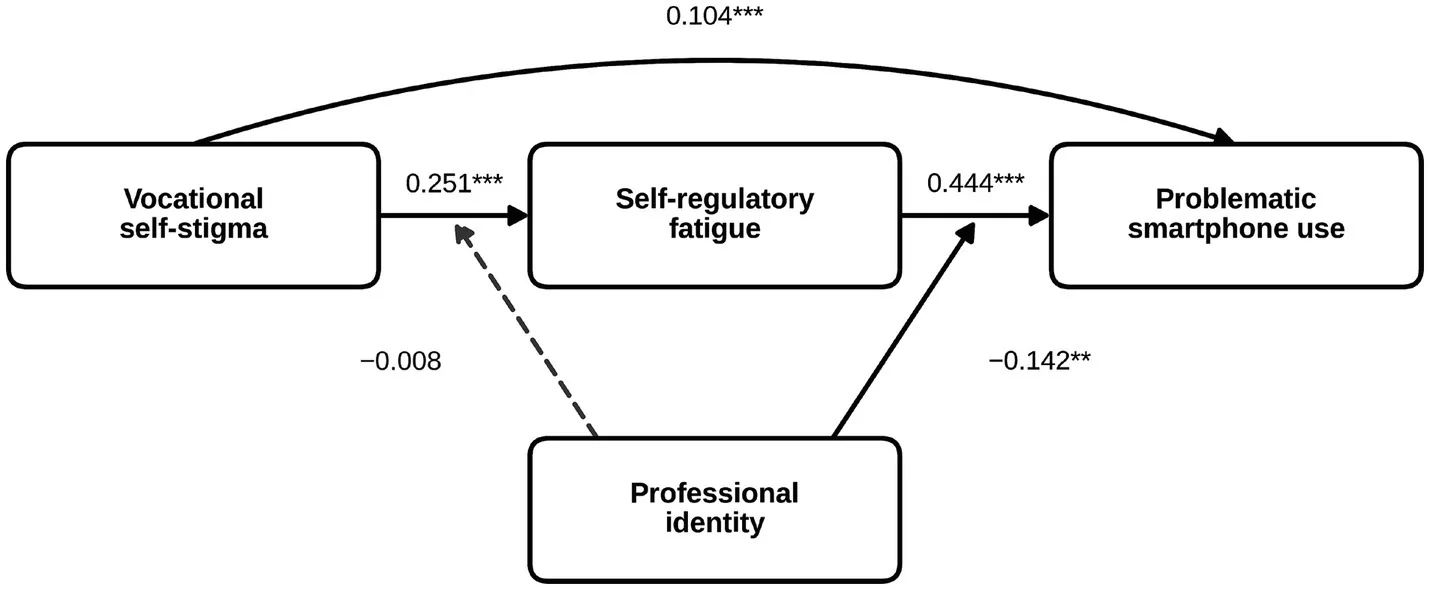
Moderated mediation model with unstandardized coefficients. Solid lines denote significant paths, and the dashed line denotes the nonsignificant first-stage interaction. Main effects of professional identity, the interaction between vocational self-stigma and professional identity in predicting problematic smartphone use (*B* = −0.026, *p* = 0.460), and the covariates are reported in Table 4. Significance tests use HC3 standard errors. **p < 0.01, ***p < 0.001.

In the equation for problematic smartphone use, fatigue was positively associated with problematic use, *B* = 0.444, *t* = 12.79, *p* < 0.001, and the interaction between fatigue and professional identity was negative, *B* = −0.142, *SE* = 0.046, *t* = −3.11, *p* = 0.002, bootstrap 95% CI [−0.228, −0.053], supporting H4. Professional identity did not moderate the direct association between self-stigma and problematic use, *B* = −0.026, *t* = −0.74, *p* = 0.460. Female students reported more problematic smartphone use than male students, *B* = 0.100, *t* = 2.49, *p* = 0.013. The model accounted for 29.5% of the variance in problematic smartphone use.

Conditional effects and conditional indirect effects appear in Table 5. The association between fatigue and problematic use was *B* = 0.542 at 1 SD below the mean of professional identity and *B* = 0.346 at 1 SD above it, whereas the association between self-stigma and fatigue barely changed across the same range (0.257 and 0.245). Accordingly, the conditional indirect effect was smaller at high than at low professional identity, 0.085, 95% CI [0.055, 0.119], vs. 0.139, 95% CI [0.103, 0.175], and the difference between them was significant, −0.054, 95% CI [−0.097, −0.010].

**Table 5:** Conditional effects and conditional indirect effects at values of professional identity.

Professional identity	Self-stigma → fatigue	Fatigue → problematic use	Conditional indirect effect	95% CI
−1 SD (2.393)	0.257	0.542	0.139	[0.103, 0.175]
Mean (3.083)	0.251	0.444	0.111	[0.086, 0.138]
+1 SD (3.774)	0.245	0.346	0.085	[0.055, 0.119]
Difference (+1 SD minus −1 SD)			−0.054	[−0.097, −0.010]

Values are unstandardized. Confidence intervals are percentile bootstrap intervals based on 5,000 resamples. The first-stage interaction coefficient was −0.008, 95% CI [−0.068, 0.053]; the second-stage interaction coefficient was −0.142, 95% CI [−0.228, −0.053].

The Johnson-Neyman analysis placed the lower boundary of significance for the association between fatigue and problematic use at a professional identity score of 4.90 (Figure 2), just below the scale maximum of 5.00; with conventional standard errors, the boundary lay just beyond the maximum, at 5.21. Because scale scores are means of six items, the highest attainable score below the maximum is 4.83, so the association was significant at every attainable score below 5.00, a range that included 98.9% of the sample. Between the 5th and 95th percentiles of professional identity (2.00 and 4.24), it declined from *B* = 0.598, 95% CI [0.487, 0.709], to *B* = 0.279, 95% CI [0.148, 0.411]. At the maximum, obtained by 15 respondents, the estimate remained positive but its confidence interval included zero, *B* = 0.171, 95% CI [−0.022, 0.364].

**Figure 2:**
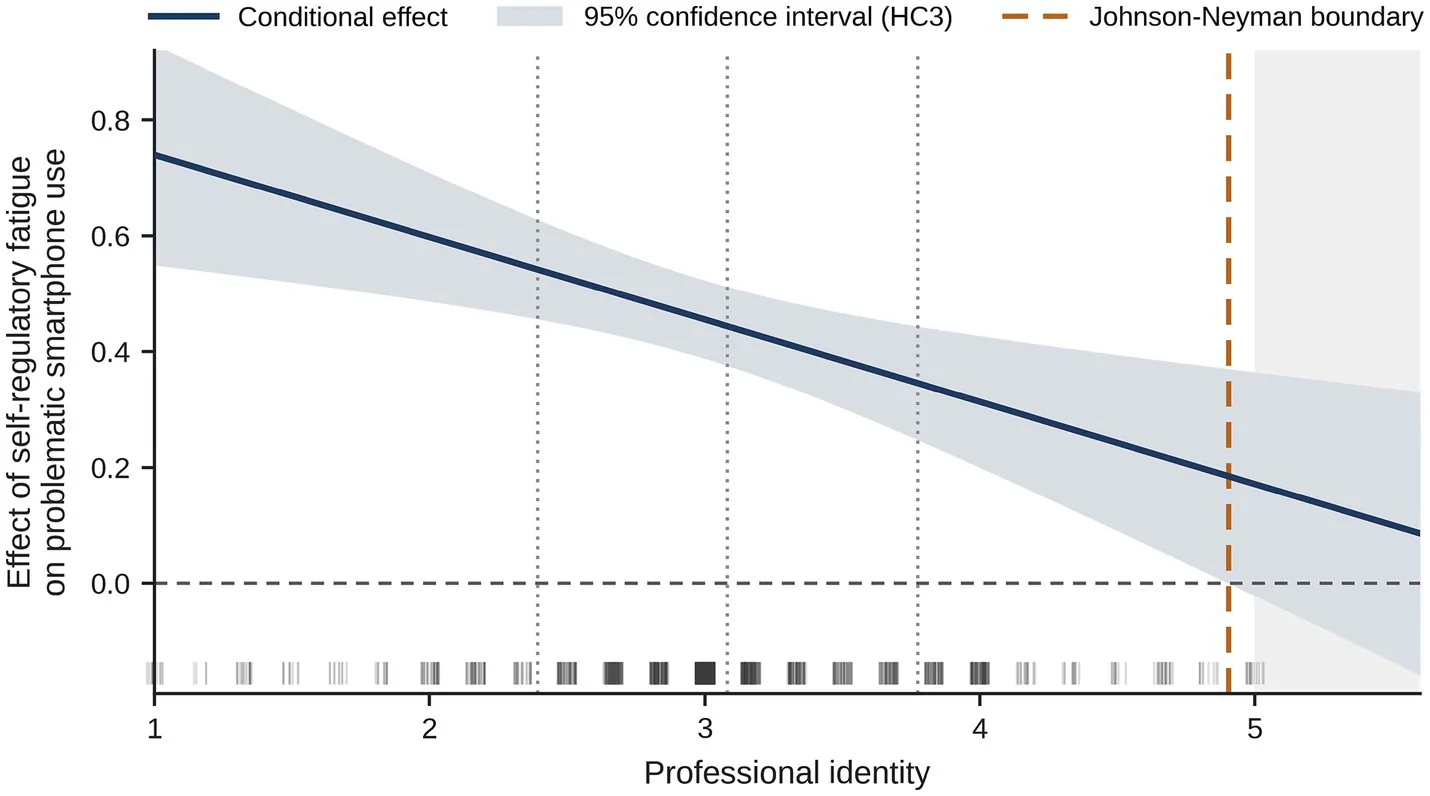
Conditional association between self-regulatory fatigue and problematic smartphone use across levels of professional identity. The shaded band shows the 95% confidence interval based on HC3 standard errors. Dotted vertical lines mark the mean of professional identity and 1 SD below and above it, and the dashed vertical line marks the Johnson-Neyman boundary (4.90); the grey region lies beyond the scale maximum of 5.00, and the upper boundary (11.78) lies outside the plotted range. Tick marks above the horizontal axis show the observed professional identity scores, jittered horizontally to reduce overlap.

### 4.4 Robustness checks

Table 2 and Figure 3 summarize the twelve specifications. The indirect effect was significant in every specification, with point estimates from 0.065 to 0.160. Omitting the energy-related item (F3), the only item representing the emotional facet of fatigue, left it unchanged (0.127). Restricting the self-stigma scale to its two self-devaluation items or to its two awareness items produced similar indirect effects, 0.110, 95% CI [0.086, 0.135], and 0.111, 95% CI [0.084, 0.139], and the concealment item alone produced 0.086, 95% CI [0.065, 0.107]. Adding the four further covariates reduced the indirect effect to 0.109, and adding response location reduced it to 0.086, 95% CI [0.063, 0.111]; both remained significant. The first-stage interaction was nonsignificant in all twelve specifications (*p* = 0.486 to 0.985). The second-stage interaction was negative in all twelve and significant in eleven; in the Zhejiang subsample it was smaller, *B* = −0.108, and its confidence interval included zero, 95% CI [−0.215, 0.015].

**Figure 3:**
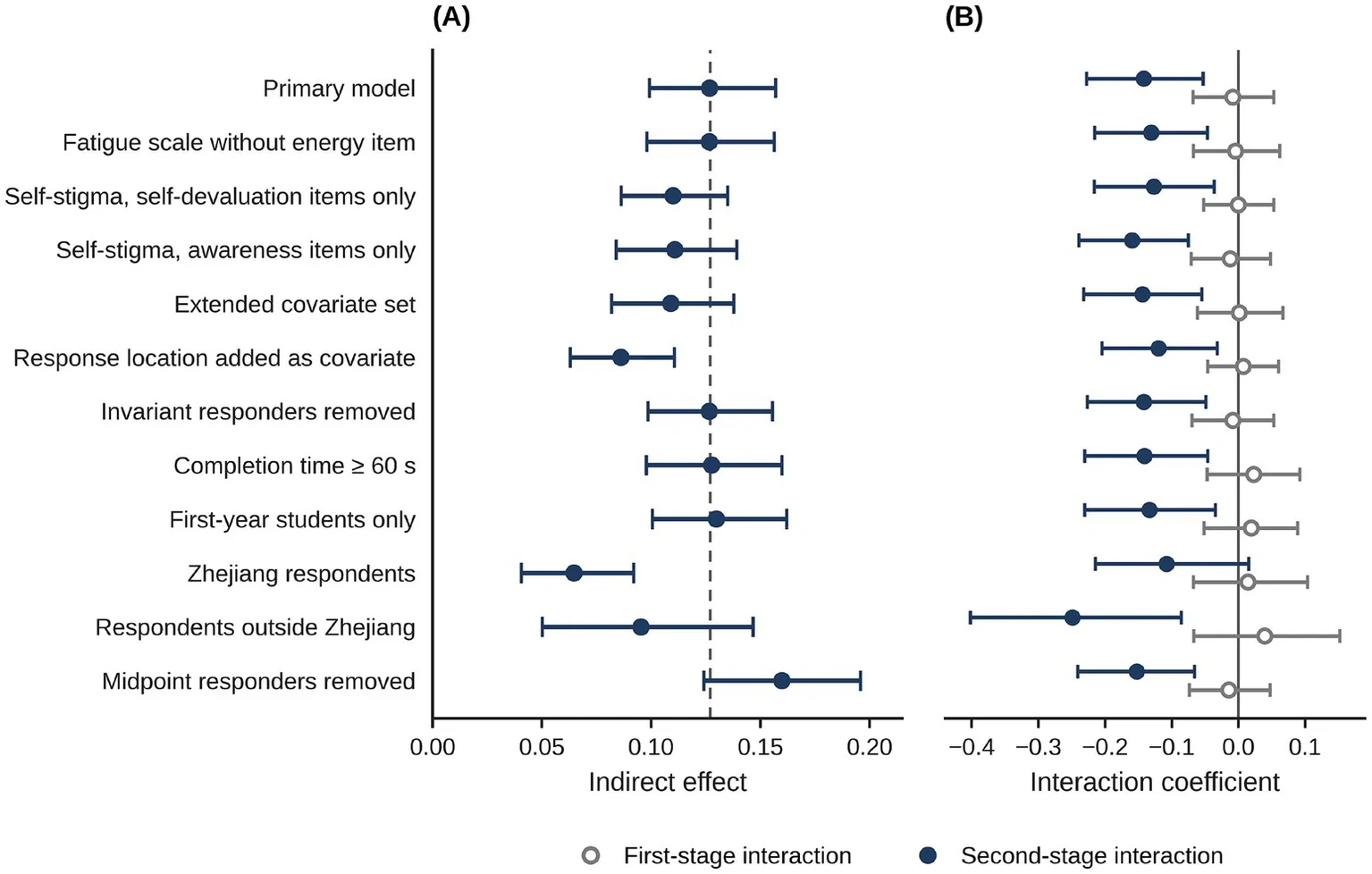
Indirect effect and interaction coefficients across the twelve analytic specifications. **(A)** Indirect effect of vocational self-stigma on problematic smartphone use through self-regulatory fatigue; the dashed line marks the primary estimate. **(B)** First-stage (open circles) and second-stage (filled circles) interaction coefficients. Error bars are 95% percentile bootstrap confidence intervals based on 5,000 resamples. Numerical results appear in Table 2.

Removing the 381 midpoint responders on professional identity strengthened the pattern. The indirect effect rose to 0.160, 95% CI [0.124, 0.196], the first-stage interaction remained close to zero (*B* = −0.014), and the second-stage interaction was slightly larger (*B* = −0.152). Internal consistency in this subsample (*α* = 0.882 to 0.908) was essentially unchanged. The results therefore did not depend on these respondents.

Respondents outside Zhejiang reported higher self-stigma (*M* = 3.29 vs. 2.71) and more problematic smartphone use (*M* = 3.31 vs. 2.68) than those within it, so part of the pooled association reflected differences between response contexts. The indirect effect remained significant within each context [Zhejiang, 0.065, 95% CI (0.041, 0.092); elsewhere, 0.095, 95% CI (0.050, 0.147)]. With fixed effects for the city of submission (eight cities with at least 20 responses and one pooled category), the indirect effect was 0.051, 95% CI [0.033, 0.071], the second-stage interaction remained significant, *B* = −0.114, 95% CI [−0.199, −0.025], whereas the first-stage interaction remained close to zero, *B* = 0.008, 95% CI [−0.045, 0.062].

A model with problematic smartphone use as the predictor and self-stigma as the outcome also produced a significant indirect effect through fatigue, 0.159, 95% CI [0.117, 0.205], against a total effect of 0.351. Cross-sectional data cannot establish which ordering holds (Maxwell and Cole, 2007).

## 5 Discussion

Among Chinese higher vocational students, those who reported more vocational self-stigma also reported more self-regulatory fatigue, and a substantial part of the association between self-stigma and problematic smartphone use was statistically indirect through fatigue. The association between fatigue and problematic use was weaker among students with a stronger professional identity, whereas the association between self-stigma and fatigue did not differ detectably across levels of professional identity.

Li W. et al. (2024) traced the association between perceived discrimination and problematic phone use in this population to negative emotions and learning burnout. The present findings add a regulatory route that is tied to students’ view of their own standing, that remained after adjustment for daily use time, family economic circumstances, career planning, and academic self-evaluations, and that persisted, at a reduced size, within cities of response. This route fits the view of stigma as an identity threat that must be managed wherever the stigmatized identity is salient (Major and O’Brien, 2005), and it is consistent with evidence that managing such threats taxes executive function and self-control in unrelated domains (Inzlicht et al., 2006; Schmader et al., 2008). Research on problematic smartphone use and research on stigma have developed largely in parallel, the former emphasizing compensatory motives and weakened inhibition (Kardefelt-Winther, 2014; Brand et al., 2019) and the latter the toll of stigma on self-regulation (Richman and Lattanner, 2014). Taken together with these literatures, the present results suggest that a devalued social identity may surface in everyday self-control problems that have no obvious connection to stigma.

Items reflecting awareness of public devaluation and items reflecting self-devaluation could not be separated empirically, and each showed an indirect association of similar size. In research on mental illness, stereotype awareness has been found to be largely unrelated to the later stages of self-stigma (Corrigan et al., 2006), and staged models locate much of the harm in the application of stereotypes to the self (Corrigan et al., 2009; Corrigan and Rao, 2012). Among Chinese higher vocational students, by contrast, perceived stigma is positively associated with self-stigma (Cui and Zhao, 2025), which fits the present result. Where devaluation is built into the structure of the education system, awareness of it may itself carry a regulatory cost, as identity threat accounts would predict (Pinel, 1999; Major and O’Brien, 2005). With two items per component, and with the awareness items and the remaining self-stigma items indistinguishable at the latent level (Section 4.1), this similarity is best treated as a question for designs that can separate the components more finely. It nonetheless cautions against assuming that only internalized devaluation matters for students’ functioning.

Students with a stronger professional identity reported less fatigue and less problematic use, but the association between self-stigma and fatigue did not differ detectably across levels of professional identity, and the first-stage interaction was small relative to the second-stage interaction (*β* = −0.01 vs. −0.09). Two *post hoc* interpretations are plausible. The buffering expected at this stage may have been offset by heightened sensitivity among students invested in their field, since people who care most about a domain are the most vulnerable to negative stereotypes that concern it (Steele, 1997; Aronson et al., 1999). Alternatively, awareness of devaluation may rest on collective representations, shared knowledge of how society regards the category (Major and O’Brien, 2005), which a student’s commitment to a field does little to change; the personal factors that the identity threat model expects to shape threat appraisal, such as group identification and stigma consciousness, refer to the stigmatized identity itself. At the second stage, students with clear occupational goals have commitments that compete with the phone when self-regulation is strained, which may explain why the moderation appeared there.

A comparable pattern has been reported for academic anxiety. Among college students, mindfulness weakened the association between self-regulatory fatigue and smartphone addiction but strengthened the association between academic anxiety and fatigue (Kong et al., 2025). In both studies, a personal resource weakened the path from fatigue to problematic use without weakening the path to fatigue. Together, the two studies suggest a hypothesis for direct testing: personal resources may matter more for how regulatory strain is expressed in behavior than for how closely a stressor is tied to that strain. Such a test could compare resources that act on goals and behavior with interventions that act on appraisal, such as reattribution training or contact-based programs that change what students believe others think of their group.

The association between fatigue and problematic use weakened as professional identity increased but remained significant for nearly all students, including those with a strong professional identity. Policy discussion of vocational education has largely favored raising the standing of vocational tracks and the occupations they serve (Billett et al., 2022; OECD, 2023). Intervention research could test whether programs that strengthen professional identity help students respond to regulatory strain in healthier ways, and whether reducing the strain itself requires addressing the devaluation of vocational education or supporting self-regulation directly. Programs that combine these elements could then be compared with single-component versions.

How far these findings extend beyond China remains open. In China, the low standing of vocational education is tied to examination-based sorting and to a long-standing preference for academic over practical learning (Guan and Blair, 2024; Ye et al., 2026), which may make the devaluation especially salient and difficult to discount. Where vocational tracks enjoy greater prestige or stronger links to skilled employment, awareness of devaluation may be weaker and its regulatory cost smaller, although the lower standing of vocational education is far from unique to China (Spruyt et al., 2015; Billett et al., 2022). The moderating role of professional identity may generalize more readily, since clear occupational goals are likely to support self-regulation in any education system. Cross-national studies that measure the perceived standing of vocational education directly could test both expectations.

Female students reported more problematic smartphone use than male students. The sex composition of vocational programs varies by field, and sex differences in prevalence have shifted over time (Lu et al., 2024), so this difference may reflect which programs students enter as much as a general sex difference. Respondents outside Zhejiang reported more self-stigma and more problematic use, and the indirect association was considerably smaller once differences between cities of response were held constant. The second-stage moderation was also weaker among respondents in Zhejiang, where it did not reach significance, although it remained significant in the full sample when differences between cities were held constant. Contexts in which vocational education is regarded differently may raise self-stigma and problematic use together. Response location is only a proxy for institutional and regional context, however, so explanations in terms of regional development or the local standing of vocational education remain speculative, and the data cannot distinguish them from unmeasured contextual differences.

This study has several limitations. The data are cross-sectional and self-reported, so the pathway describes statistical associations and cannot establish temporal order; a model with the order of self-stigma and problematic use reversed also yielded a significant indirect effect. Longitudinal and experience-sampling designs could test whether self-stigma, fatigue, and problematic use reinforce one another over time, and behavioral records of smartphone use would reduce reliance on self-report. Common method variance cannot be excluded, although it tends to deflate interaction effects instead of creating them (Siemsen et al., 2010). The scales were abbreviated to five or six items; the fatigue measure, for example, used 5 of the 16 items of the Chinese Self-Regulatory Fatigue Scale, and the smartphone measure 6 of the 17 items of the Mobile Phone Addiction Index. Reliability and factor structure were sound, but the findings pertain to the core content each short form retained, and the cognitive and emotional facets of self-regulatory fatigue were each represented by a single item. Data quality also warrants caution. Many respondents finished quickly or gave identical answers within a scale, and more than a quarter chose the midpoint on every professional identity item; removing fast, invariant, and midpoint responders left the main results unchanged, but insufficient effort can bias survey associations in either direction (Huang et al., 2015). The sample was a convenience sample, concentrated in Zhejiang and dominated by first-year students, although the pattern held in the first-year subsample. Students were nested within classes and five colleges, but institutional and class membership were not recorded, so nesting could be approximated only through fixed effects for the city of response; five colleges would in any case be too few for multilevel modeling (McNeish and Stapleton, 2016).

Professional identity was the only identity assessed as a potential resource. Among Chinese higher vocational students, self-stigma is associated with weaker identification with vocational education (Cui and Zhao, 2025), and among prisoners, ingroup ties and identity centrality relate to self-stigma in opposite ways (Traversa et al., 2026). Measuring these forms of group identification alongside professional identity would show whether any of them bears on the first stage of the pathway. Because depleted members of subordinate groups tend to perceive less prejudice (Genç Turhan and Kuşdil, 2025), such work could also examine whether the regulatory strain associated with self-stigma makes devaluation harder to recognize.

## 6 Conclusion

Among Chinese higher vocational students, vocational self-stigma was statistically linked to problematic smartphone use through self-regulatory fatigue, and professional identity was associated with a weaker link between fatigue and problematic use but not with a weaker link between self-stigma and fatigue. Whether strengthening professional identity, reducing the devaluation of vocational education, or supporting students’ self-regulation can interrupt this pathway is a question for longitudinal and intervention research.

## Data Availability

The raw data supporting the conclusions of this article will be made available by the authors, without undue reservation.

## Supplementary material

SUPPLEMENTARY TABLE S1 Full item set.

SUPPLEMENTARY TABLE S2 Analytic specifications used in the robustness analyses.
