# Supplementary material for: Vocational self-stigma and problematic smartphone use: self-regulatory fatigue as an indirect pathway and professional identity as a second-stage moderator
Source: Front Psychol. 2026 Oct 15;17:1942617. doi: 10.3389/fpsyg.2026.1942617 (PMC13716854; doi:10.3389/fpsyg.2026.1942617)
Supplement: Supplementary file 1 [file Supplementary_file_1.docx]

**Supplementary Material**

**Supplementary Table S1.** Full item set

All items were answered on a five-point Likert scale from 1 (strongly disagree) to 5 (strongly agree). Items were administered in Chinese; the English wording below is a translation provided for reference and was not used in data collection. Reverse-scored items were recoded before analysis. Item F3 was omitted in the robustness specification without the energy item (Supplementary Table S2). Content labels describe what each item assesses.

| Item | English translation | Original Chinese wording | Content |
| --- | --- | --- | --- |
| **Vocational self-stigma** | | | |
| S1 | I sometimes worry that others look down on me because of my institution. | 我有时会担心，别人因为我的学校而看低我 | Awareness of public devaluation |
| S2 | I sometimes think less of myself because of my status as a higher vocational student. | 我有时会因为“高职生”这个身份而看轻自己 | Self-devaluation |
| S3 | I feel I am not as good as students at general universities. | 我觉得自己不如普通高校的学生 | Self-devaluation |
| S4 | I am reluctant to bring up my institution with others. | 我不太愿意主动向别人提起自己的学校 | Concealment |
| S5 | I think many people in society hold prejudiced views about higher vocational students. | 我觉得社会上很多人对“高职生”存在偏见 | Awareness of public devaluation |
| **Self-regulatory fatigue** | | | |
| F1 | Lately I find it hard to concentrate for long. | 我最近很难长时间集中注意力 | Cognitive |
| F2 | I am self-disciplined in daily life. (reverse-scored) | 我在日常生活中很自律 | Behavioral |
| F3 | I often feel I lack the energy to see tasks through. | 我常常感到精力不足，难以坚持完成任务 | Emotional |
| F4 | I tend to give up on things partway. | 我做事情容易半途而废 | Behavioral |
| F5 | I know I should persist, but sometimes I cannot. | 我明知道应该坚持，但有时做不到 | Behavioral |
| **Problematic smartphone use** | | | |
| P1 | I often use my phone for longer than I intended. | 我使用手机的时间常常超过原本的计划 | Loss of control |
| P2 | I feel uneasy or anxious when I am not using my phone. | 不使用手机时，我会感到不自在或焦虑 | Anxiety and craving |
| P3 | I use my phone to escape from real-life problems. | 我会通过玩手机来逃避现实中的问题 | Withdrawal and escape |
| P4 | I have tried to cut down my phone use without much effect. | 我曾尝试减少使用手机，但效果不明显 | Loss of control |
| P5 | My phone use has affected my studies or daily life. | 手机使用已经对我的学习或生活产生影响 | Productivity loss |
| P6 | I check my phone frequently without meaning to. | 我会不自觉地频繁查看手机 | Anxiety and craving |
| **Professional identity** | | | |
| I1 | I feel confused about my future employment and career development. (reverse-scored) | 我对未来的就业与职业发展感到迷茫 | Stability of occupational goals |
| I2 | I am unsure whether I am suited to my current field of study. (reverse-scored) | 我不确定自己是否适合现在所学的专业 | Appraisal of ability |
| I3 | I have genuinely chosen an occupation related to my field as my goal, and I am willing to make my best effort. | 我从心底选择将与本专业相关的职业作为目标，并愿意付出最大的努力 | Career decision |
| I4 | I believe I am suited to an occupation related to my field. | 我相信自己适合从事与本专业相关的职业 | Appraisal of ability |
| I5 | I have a fairly clear sense of direction about my future occupation. | 我对未来从事的职业有比较清晰的方向 | Stability of occupational goals |
| I6 | I like my field of study and endorse its value. | 我喜欢本专业，并且认同自己所学专业的价值 | Vocational interest |

**Supplementary Table S2.** Analytic specifications used in the robustness analyses

All specifications used regression models corresponding to PROCESS Models 4 and 59, with percentile bootstrap confidence intervals based on 5,000 resamples. Specifications 1 to 12 correspond to the rows of Table 2 in the main text. Item codes refer to Supplementary Table S1.

| Specification | Variation from the primary model | Purpose | n |
| --- | --- | --- | --- |
| 1. Primary model | All items; sex and year of study as covariates | Test of the hypothesized model | 1,352 |
| 2. Fatigue scale without energy item | Item F3 omitted from the fatigue scale | Checks whether results depend on the energy-related item | 1,352 |
| 3. Self-stigma, self-devaluation items only | Self-stigma scored from items S2 and S3 | Examines the self-application component alone | 1,352 |
| 4. Self-stigma, awareness items only | Self-stigma scored from items S1 and S5 | Examines the awareness component alone | 1,352 |
| 5. Extended covariate set | Adds daily smartphone use time, career planning clarity, family economic circumstances, and self-rated academic performance | Adjusts for correlates that may overlap with the study constructs | 1,352 |
| 6. Response location added as covariate | Adds response location (Zhejiang vs. other provinces) | Adjusts for differences between response contexts | 1,352 |
| 7. Invariant responders removed | Excludes respondents who gave the same answer to all 22 items | Data quality | 1,342 |
| 8. Completion time ≥ 60 s | Excludes respondents who finished in under 60 s | Data quality | 1,140 |
| 9. First-year students only | Restricts the sample to first-year students | Sample composition | 1,065 |
| 10. Zhejiang respondents | Restricts the sample to responses from Zhejiang | Response context | 1,066 |
| 11. Respondents outside Zhejiang | Restricts the sample to responses from other provinces | Response context | 286 |
| 12. Midpoint responders on professional identity removed | Excludes respondents who chose the midpoint on every professional identity item | Moderator concentrated at a single value | 971 |
| *Additional analyses (not counted among the twelve specifications)* | | | |
| Concealment item only | Self-stigma scored from item S4 | Examines concealment alone | 1,352 |
| Reverse ordering | Problematic smartphone use as predictor and self-stigma as outcome, with fatigue as mediator | Examines the alternative ordering | 1,352 |
| City fixed effects | Adds indicators for the city from which the questionnaire was submitted (eight cities with at least 20 responses and one pooled category) to both equations | Absorbs mean differences between response contexts, as institutional membership was not recorded | 1,352 |
